# Supplementary material for: Nano DNA Vaccine Encoding Toxoplasma gondii Histone Deacetylase SIR2 Enhanced Protective Immunity in Mice
Source: Pharmaceutics. 2021 Sep 29;13(10):1582. doi: 10.3390/pharmaceutics13101582 (PMC8538992; doi:10.3390/pharmaceutics13101582)
Supplement: Supplementary file 1 [file pharmaceutics-13-01582-s001.zip › pharmaceutics-1366740-SI.pdf]

pharmaceutics

MDPI

Article

Nano DNA Vaccine Encoding *Toxoplasma gondii* Histone Deacetylase SIR2 Enhanced Protective Immunity in Mice

Zhengqing Yu, Yujia Lu, Wandi Cao, Muhammad Tahir Aleem, Junlong Liu, Jianxun Luo, Ruofeng Yan, Lixin Xu, Xiaokai Song and Xiangrui Li *

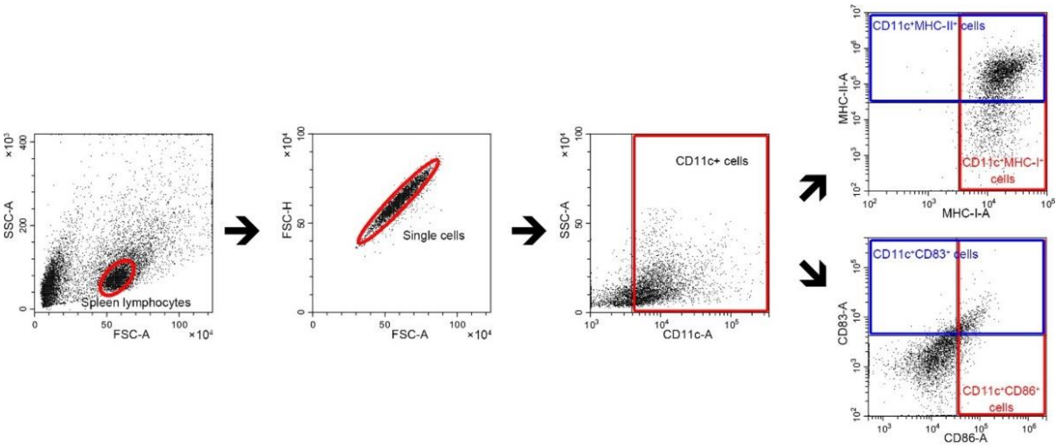

Figure S1. The gating strategy of CD11c⁺CD83⁺ cells, CD11c⁺CD86⁺ cells, CD11c⁺MHC-I⁺ cells, and CD11c⁺MHC-II⁺ cells in spleen. CD11c⁺ cells, CD11c⁺CD83⁺ cells, CD11c⁺CD86⁺ cells, CD11c⁺MHC-I⁺ cells, and CD11c⁺MHC-II⁺ cells were gated according to fluorescence minus one (FMO) controls.

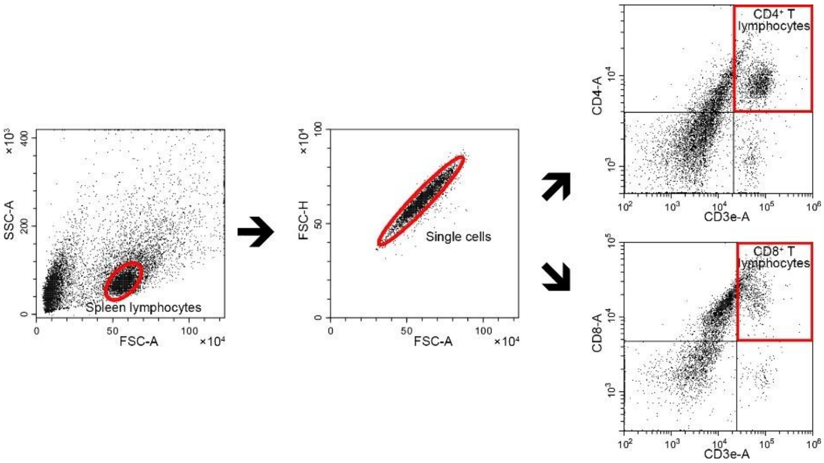

Figure S2. The gating strategy of CD3e⁺CD4⁺ and CD3e⁺CD8⁺ cells in spleen. CD3e⁺CD4⁺ and CD3e⁺CD8⁺ cells were gated according to the FMO controls.
